# Viral expression associated with gastrointestinal adenocarcinomas in TCGA high-throughput sequencing data

**DOI:** 10.1186/1479-7364-7-23

**Published:** 2013-11-27

**Authors:** Daria Salyakina, Nicholas F Tsinoremas

**Affiliations:** 1 Center for Computational Science, University of Miami, 1120 NW 14 St, Miami, FL 33136, USA; 2 Department of Medicine, Miller School of Medicine, University of Miami, 1120 NW 14 St, Miami, FL 33136, USA

**Keywords:** Cancer, Papilloma virus, Herpes virus

## Abstract

**Background:**

Up to 20% of cancers worldwide are thought to be associated with microbial pathogens, including bacteria and viruses. The widely used methods of viral infection detection are usually limited to a few *a priori* suspected viruses in one cancer type. To our knowledge, there have not been many broad screening approaches to address this problem more comprehensively.

**Methods:**

In this study, we performed a comprehensive screening for viruses in nine common cancers using a multistep computational approach. Tumor transcriptome and genome sequencing data were available from The Cancer Genome Atlas (TCGA). Nine hundred fifty eight primary tumors in nine common cancers with poor prognosis were screened against a non-redundant database of virus sequences. DNA sequences from normal matched tissue specimens were used as controls to test whether each virus is associated with tumors.

**Results:**

We identified human papilloma virus type 18 (HPV-18) and four human herpes viruses (HHV) types 4, 5, 6B, and 8, also known as EBV, CMV, roseola virus, and KSHV, in colon, rectal, and stomach adenocarcinomas. In total, 59% of screened gastrointestinal adenocarcinomas (GIA) were positive for at least one virus: 26% for EBV, 21% for CMV, 7% for HHV-6B, and 20% for HPV-18. Over 20% of tumors were co-infected with multiple viruses. Two viruses (EBV and CMV) were statistically significantly associated with colorectal cancers when compared to the matched healthy tissues from the same individuals (*p* = 0.02 and 0.03, respectively). HPV-18 was not detected in DNA, and thus, no association testing was possible. Nevertheless, HPV-18 expression patterns suggest viral integration in the host genome, consistent with the potentially oncogenic nature of HPV-18 in colorectal adenocarcinomas. The estimated counts of viral copies were below one per cell for all identified viruses and approached the detection limit.

**Conclusions:**

Our comprehensive screening for viruses in multiple cancer types using next-generation sequencing data clearly demonstrates the presence of viral sequences in GIA. EBV, CMV, and HPV-18 are potentially causal for GIA, although their oncogenic role is yet to be established.

## Background

Viruses may be more commonly associated with malignant diseases than previously considered [1]. Reported associations do not always mean that a virus is a direct cause of the cancer; they can be the result of contamination, viral infection without causal involvement (‘passenger’), and an indirect or direct causal relationship. Regardless of the causal relationship, viruses may have significant clinical implications in human cancers through contribution to dramatic changes in the microenvironment and immunosurveillance.

The main strategies to detect and type various viruses in cancers usually address individual protein biomarkers, serological tests, or DNA/RNA detection of one or a few viruses at a time. The major disadvantage of these strategies is failure to detect viruses not previously known to be associated with a particular cancer type. In this report, we introduce a new and substantially different way of addressing this problem by utilizing next-generation sequencing (NGS) data to detect both human and non-human nucleic acids in tumor specimens. This approach does not require any prior knowledge of viruses involved and can identify all known viral genomes. NGS provides the opportunity to detect viral transcripts with high sensitivity in the host tissue at frequencies less than 1 RNA molecule in 1 million [2]. Whole genome or transcriptome tumor sequencing data provides a unique resource for the development of new and powerful methodologies to detect and characterize viruses in cancers.

‘Computational subtraction’ is the general concept for detecting infectious agents in the host NGS material [3]. During this procedure, human and artifact sequences are removed from the NGS data and the remaining sequences are aligned to bacterial or viral references from existing databases. A few groups have implemented computational subtraction procedures for this purpose [4,5]. To date, NGS data was successfully used for virus identification in human papilloma virus (HPV)-associated squamous cell carcinomas [6-9] and hepatitis B virus (HBV)-mediated hepatocellular carcinomas [5], while other cancer types and viruses largely remain out of the picture.

In this study, we present a comprehensive screening for viruses in NGS data of nine common cancers in 1,007 patients, using The Cancer Genome Atlas (TCGA) data. TCGA is a joint project of the National Cancer Institute (NCI) and the National Human Genome Research Institute (NHGRI). The goal of the TCGA project is to collect and systematically explore the entire spectrum of genomic changes involved in more than 20 types of human cancers. Comprehensive genomic characterization has been published for three out of nine cancers studied so far (lung squamous cell carcinoma, colon, and rectum adenocarcinoma) [10,11]. Colon and rectum adenocarcinoma were shown to belong to one cancer type based on their molecular profiles. In addition to the screening for viruses, we perform association analysis of identified viruses with tumor vs. paired non-malignant tissue from the same patients in order to determine whether the presence of a virus is significantly associated with tumors and not the normal cell types.

## Results and discussion

### Screening for viruses

**Figure 1:**
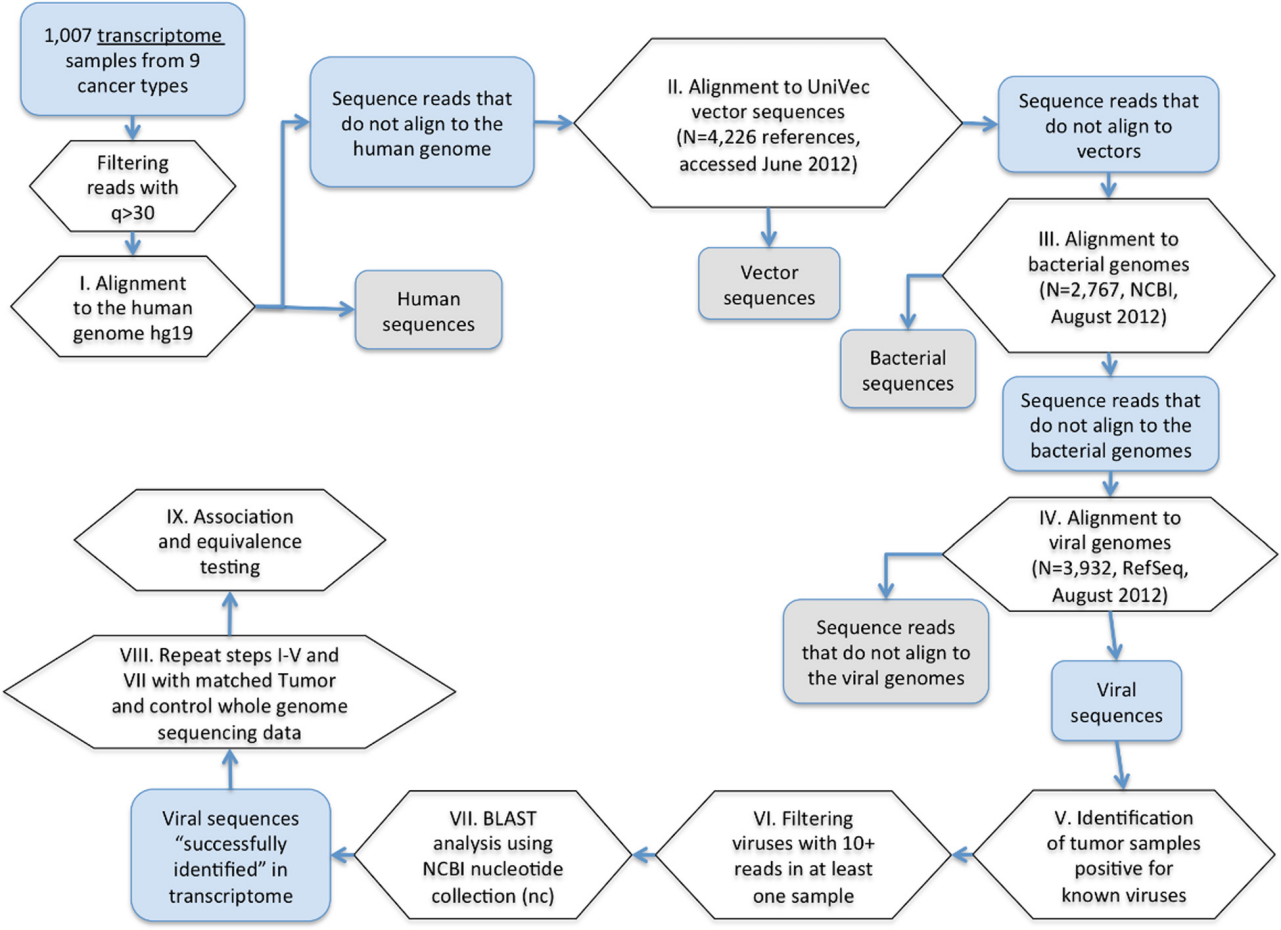
**Data analysis flowchart.** Before step I, sequencing reads with phred-like quality scores *q* < 30 were removed. *N* in steps II, III, and IV reflects the number of reference sequences from corresponding databases. For the alignment in steps II, III, and IV, we combined reference fasta files into ‘supergenomes’ including vector sequences, bacterial genomes, and viral genomes, respectively. Each individual reference sequence in the ‘supergenome’ was treated as a chromosome. All supergenome reference files were indexed before alignment steps.

Screening for viruses is an essential step in the continuum of research that is expected to lead to new treatment strategies in patients with virus-positive tumors. In this study, we performed the systematic screening for potentially oncogenic viruses in nine cancer types from TCGA (Table 1), most of which were not previously known to be associated with viruses or have controversial reports in this regard [12,13]. After subtraction of non-viral sequences, unaligned fragments were used in virus identification (Figure 1). Sequences of Epstein-Barr virus (EBV), cytomegalovirus (CMV), roseola virus (HHV-6B), Kaposi’s sarcoma-associated human virus (KSHV), and human papilloma virus type 18 (HPV-18) were identified in transcriptomes of three gastrointestinal adenocarcinomas (GIA): stomach, rectum, and colon adenocarcinomas (STAD, READ, and COAD, respectively). In total, 83% of the viral reads mapped to the known coding regions. The remaining six cancer types (Table 1) were virus negative according to our computational pipeline. Table 2 represents a summary for the sequencing data in the GIA samples and matched controls.

**Table 1:** Sample size for available DNA and RNA sequencing data in primary tumors and paired control samples

**Cohort**	**Primary tumor**		**Matched controls**	
			**Blood**	**Solid tissue**
	**RNA**	**DNA**	**DNA**	**DNA**
AML, acute myeloid leukemia	123	nt	nt	nt
COAD, colon adenocarcinoma	194	77	56	16
KIRK, kidney renal clear carcinoma	132	nt	nt	nt
KIRP, kidney renal papillary cell carcinoma	15	nt	nt	nt
LUAD, lung adenocarcinoma	58	nt	nt	nt
LUSK, lung squamous cell carcinoma	151	nt	nt	nt
READ, rectum adenocarcinoma	71	40	35	4
STAD, stomach adenocarcinoma	57	3	0	2
UCEC, uterine corpus endometrioid carcinoma	157	nt	nt	nt

Control samples were derived from either whole blood or adjacent normal tissue (not both) from the same patient. nt, not tested.

**Table 2:** Total number of next-generation sequencing reads/fragments available for gastrointestinal cancers organized by cancer and tissue type

**Cohort**	**Statistics**	**Primary tumor**	**Primary tumor**		**Blood**		**Solid tissue**	
		**RNA**	**DNA**	**Human genome coverage**^**a**^	**DNA**	**Human genome coverage**^**a**^	**DNA**	**Human genome coverage**^**a**^
COAD	Min	5,244,743	60,872,080	0.47	64,637,478	0.50	148,341,394	1.18
	Mean	26,127,635	205,217,167	1.63	182,959,915	1.45	224,838,391	1.77
	Median	27,424,762	196,408,988	1.57	193,657,350	1.49	196,072,876	1.57
	Max	64,021,517	1,162,469,344	9.43	354,790,740	2.85	429,443,164	3.36
READ	Min	19,665,974	89,943,982	0.70	90,122,736	0.70	95,061,666	0.75
	Mean	26,782,703	225,818,442	1.79	214,787,544	1.71	150,549,985	1.19
	Median	27,121,702	227,226,239	1.81	224,045,358	1.80	159,027,517	1.26
	Max	33,338,326	461,153,444	3.68	367,295,986	2.93	189,083,240	1.47
STAD	Min	127,157,036	259,701,502	2.08	NA	NA	418,702,808	3.34
	Mean	155,138,810	300,668,630	2.40	NA	NA	448,318,606	3.58
	Median	155,490,548	280,015,346	2.23	NA	NA	448,318,606	3.58
	Max	193,445,130	362,289,042	2.88	NA	NA	477,934,404	3.82

Paired reads are counted as two single fragments. COAD, colon adenocarcinoma; READ, rectum adenocarcinoma; STAD, stomach adenocarcinoma; NA, no data available. ^a^Average coverage was calculated as the number of reads multiplied by the average read length (51 nt) and divided by the corresponding genome length, divided by two for diploid genome.

### Viruses in gastrointestinal adenocarcinomas

**Figure 2:**
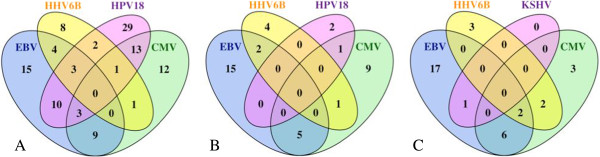
**Counts of tumors with viral co-infection. (A)** In the COAD, **(B)** in the READ, and **(C)** in the STAD. Tumors were considered virus positive when viral sequences were identified in the tumor transcriptome or genome.

EBV, CMV, and HHV-6B were detected in all three GIA, while HPV-18 was detected in colorectal cancers only. In total, 189 (58.7%) GIA samples were infected with at least one virus: 83 (25.8%) with EBV, 67 (20.8%) with CMV, 22 (6.8%) with HHV-6B, and 64 (19.8%) with HPV-18 (Figure 2). One STAD RNA sample was KSHV positive with 115 sequence reads mapping to 3,280 bases of the viral genome (Additional file 1: Table S1). During the review of this paper, another group published a very similar study including 3,775 tumors from TCGA [14]. However, in their computational pipeline, Khoury with colleagues did not detect any viral sequences in 138 COAD and 66 READ samples. In addition, they only reported four EBV-positive samples in the STAD. Khoury et al. used a different sequence aligner (MOSAIK) and more stringent cutoff for the viral gene expression detection. Although no explicit cutoff is stated in the paper [14], the VirusSeq pipeline [5] implemented in their study uses 1,000 reads as a cutoff for virus detection. In our case, the number of identified reads was far below 1,000 for all transcriptomes, except two EBV-positive STAD tumors. The probable reason for using such a high cutoff in VirusSeq pipeline is the high number of false-positive results due to the homology of the contaminating vector and bacterial sequences. In our pipeline, adding steps II and III, in which these sequences are subtracted, alleviates this problem, reducing the number of unaligned RNA-seq reads by 19% on average before aligning to the virus reference. In our experience, these two steps are essential since they eliminate false-positive findings and increase specificity of the method. We included here 57 STAD samples, not 71 as in [14]. To our knowledge, 14 additional patients reported in [14] have withdrawn their consent to participate in TCGA study (as of November 1, 2012).

All viruses identified in GIA are ubiquitous in human population. The first encounter with EBV, CMV, and HHV-6B infection usually happens in early childhood and results in latent lifelong infection with a prevalence of over 90% in adults [15-21]. Similarly, KSHV infects 7.2% and 49% of the population depending on the geographical region [22-24]. At the same time, HPV is the most common sexually transmitted infection with a lifelong prevalence of 71%, although unlike herpes virus infections, about 90% of HPV infection is cleared within 2 years without any consequences [25].

EBV, HPV-18, KSHV, and CMV have been linked to multiple cancers and potentially are oncogenic in GIA. EBV has been accepted as an infective agent of gastric and colorectal carcinomas. Nearly 10% of all gastric cancers [26-30] and up to 30% of colorectal carcinomas [31-35] have been found to be EBV-infected monoclonal epithelial cells. In TCGA, 45.6% of STAD, 22.6% of COAD, and 31% of READ samples were EBV positive [36,37] (Figure 2). Multiple studies have also shown significant association of high-risk HPV types with colorectal cancers with infection rate of up to 84% [38-41]. The detected frequency of HPV-18 in TCGA was substantially lower: 31% in COAD and 0.4% in READ. Differences in frequencies between previously reported and our studies can result from multiple factors, such as varying sensitivity of detection methods used as well as population demographics. For instance, EBV positivity in STAD has been reported to be higher in males, young subjects, non-antral subsites, diffuse-type histology, and in studies from the Americas [27]. Given the sample size of the STAD cohort and the number of viruses, no epidemiological analyses were done. COAD and READ combined provided a sufficient sample size for association testing with clinical and demographic variables (Additional file 1: Tables S2–S12). No association with gender, age at initial diagnosis, histological type or ‘M’ and ‘N’ staging was found. Nominal *p* values are shown in Table 3. After correction for multiple testing, the only association of HPV-18 infection with anatomic subdivision was statistically significant. HPV-18 was predominantly associated with tumors located in the cecum and ascending colon. No large epidemiological studies for HPV-associated COAD are available thus far to compare these results to.

**Table 3:** **Nominal *p *** **values for association testing of virus with clinical and demographic phenotypes**

**Phenotype**	**HPV-18**	**EBV**	**CMV**	**HHV-6B**
Gender	1.00	1.00	1.00	0.54
Age at initial diagnosis	0.22	0.51	0.07	0.26
Anatomic subdivision	5.0E − 05*	0.23	0.06	0.02
Histological type	0.39	0.52	0.82	0.55
History of colon polyps	3.0E − 03	0.77	0.22	0.83
Pathologic M	0.84	0.32	0.21	1.00
Pathologic N	0.59	0.11	0.58	0.13
Pathologic T	0.33	0.03	0.09	0.48
Stage	0.89	0.01	0.12	0.64

*p value is significant after Bonferroni correction at *α* = 0.05.

CMV is also capable of transforming mammalian cells through various pathways [42] and has been linked to colorectal cancer, although available evidence is scarce [43]. KSHV causes Kaposi’s sarcoma [44] and, to our knowledge, has not yet been associated with gastric adenocarcinomas. This may be a special case of STAD that needs further investigation.

### Co-infection with multiple viruses

A substantial proportion of GIA was co-infected with multiple viruses: 46 (23%) COAD, 11 (19.3%) STAD, and 9 (12.7%) READ. Figure 2 shows how many tumors were co-infected. These multiple viruses may either co-exist in the same cancer cells or populate different cell types that compose or infiltrate the tumor. Szostek et al. suggests that co-infection with HHVs, especially CMV and EBV, may increase probability of the HPV-16 integration into the host genome during cervical cancer tumorigenesis [45]. Similar mechanisms may be involved with EBV, CMV, and HPV-18 in colorectal adenocarcinomas and need to be tested in future studies. Alternatively, some of the identified viruses can also preferentially infect cancer cells, taking advantage of the impaired immune environment of the tumors.

### Virus quantification

**Figure 3:**
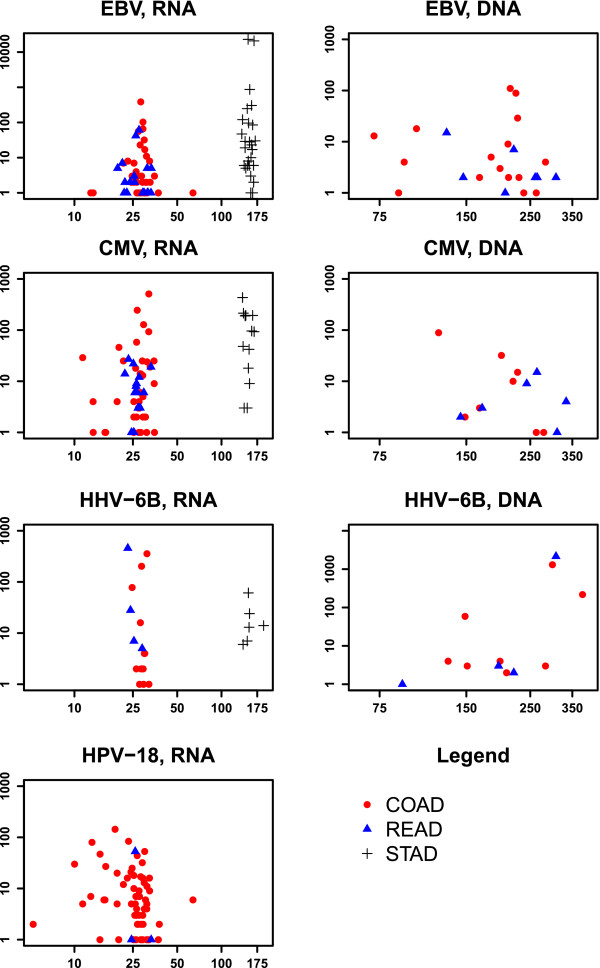
**Number of sequencing reads mapped to virus reference versus the total number of reads.** The *X*-axis shows total RNA or DNA fragments (short sequencing reads) in millions, and the *Y*-axis shows the number of fragments mapped to the particular virus, as stated in the plot subtitles. Each data point represents one tumor. HPV-18 DNA was not detectable in the whole genome sequencing data. COAD are depicted as read circles, READ as blue triangles, and STAD as black plus signs.

The proportion of viral transcriptome reads relative to the total number of reads in the GIA ranged from 6 × 10^−9^ (HHV-6B) to 2 × 10^−5^ (EBV). The majority of tumors did not have a sufficient number of reads to cover the whole viral genome or to allow transcript quantification (see Additional file 1: Table S1 and Additional file 2). The number of viral sequence reads detected in tumor transcriptomes varied from a single fragment to tens of thousands of fragments per sample, with a strong skew towards low counts (Figure 3). Only five or less viral sequence fragments were detected in 49% of the transcriptomes and only one fragment in 21%. However, our follow-up Basic Local Alignment Search Tool (BLAST) analysis, described in the ‘Methods’ section, demonstrated that even detection of a single short read may be sufficient to ensure unambiguous viral detection, confirming high specificity of sequencing alignment [8].

The estimated viral load for EBV, CMV, and HHV-6B in GIA was less than one viral copy per cell (vc/c) in all cases with a maximum of 0.72 vc/c (HHV-6B) (Additional file 1: Table S1). The latter is equivalent to one viral genome per 1.39 human cells. This data supports the hypothesis that only a small proportion of tumor cells had a virus. The viral DNA abundance for EBV and CMV correlated with the proportion of total viral RNA reads (Additional file 3). Because no genomic data was available for KSHV-positive tumor and no HPV-18 was detectable in genomic DNA from tumors or normal cells, no viral load for these viruses could be calculated. The HPV-18 genome (7,857 nt for RefSeq ID: NC_001357) is 20 to 30 times smaller than the HHV (162,114–235,646 nt). The HPV-18 DNA quantity must have been below the detection limit at the available sequencing depth. The lowest detection threshold for NGS studies is limited to one sequence read aligning to the target genome. As a result, the probability for detecting a viral sequence in the host NGS data will be proportional to the target sequence length, the viral load, and the sequencing depth. Given the HPV-18 size and average genome sequencing depth of GIA, the hypothetical average detection limit would be above 0.0047 vc/c (standard deviation (SD) = 0.0023), equivalent to 1 virus in 261 human cells (SD = 133). This estimation does not take into account possible data loss due to disproportional filtering of highly polymorphic viral sequences through an unaccepted number of mismatches and homopolymeric and repetitive regions. Thus, the viral load is most likely underestimated here. Currently, there is no clear consensus on the minimum viral load indicative for the virus causality in neoplasm. On one hand, viral genome abundance and active expression of viral oncogenes are broadly believed to indicate much greater viral involvement in disease than the silent presence of viral genome. On the other hand, according to the ‘hit-and-run’ mechanism, transient acquisition of viral genome may be sufficient to induce malignant conversion [46]. In the hit-and-run scenario, viruses may get partially or completely lost after they cause permanent damage to the host cell and are no longer necessary for the maintenance of the malignant state. The most reliable estimates of viral load from the literature are related to HPV-associated tumors. The quantitative PCR experiments report several HPV-18 copies per cell [8,47]. However, Yoshida et al. suggested that very early HPV-18 DNA integration may result in lower copy numbers in cervical adenosquamous carcinoma (1.50–0.89 vc/c), leading to a more aggressive transformation with greater chromosomal instabilities, higher growth rates, and rapid progression [47].

### Virus association with tumors

Next, we tested if identified viruses were mostly found in tumors compared to the non-malignant matched tissue from the same individuals. One of the limitations of this study is the absence of transcriptome data for matched non-malignant tissues. Before association analysis, we explored if transcriptome and genome sequencing data can be pooled for tumors and compared to genomic data from non-malignant tissues. According to our results, tumor DNA and RNA did not show statistically equivalent rates of virus positivity (Table 4). For this reason, only DNA data from both tumors and non-malignant matched controls were utilized for association analysis. A sufficient number of whole genome sequencing data was available for three viruses: EBV, CMV, and HHV-6B in COAD and READ only. Since COAD and READ are genetically identical [11], we combined the two into colorectal adenocarcinomas (CRAD) to achieve a higher sample size for association analysis. Our results indicate that EBV and CMV were significantly associated with CRAD vs*.* matched non-malignant specimens with *p* = 0.0022 (*p*_corrected_ = 0.02) and *p* = 0.0034 (*p*_corrected_ =0.03), respectively (Table 5). Since testing was done in three tissue types: solid tumor, solid non-malignant adjacent tissue, and whole blood, these associations may reflect tissue-specific infection. Thus, we investigated if viral rates differ between tissue types in controls. All controls positive for EBV and CMV were blood samples, and none of the tested solid tissue controls had these two viruses (Table 5). In contrary, HHV-6B was identified predominantly (80%) in solid non-malignant tissue and not blood, although association was not significant after correction for multiple testing (*p*_corrected_ = 0.07). These findings may reflect the fact that EBV and CMV are associated with tumors, while HHV-6B is associated with the colorectal tissue. EBV, CMV, and HPV-18 are capable to infect different types of skin cells, endothelium, or mucus membranes and are plausible infections in the histological context of GIA. While the oncogenic nature of CMV is still debatable [42], EBV and HPV-18 are potentially causal in GIA because both viruses encode oncoproteins, which are able to transform human cells [30,39,48]. HHV-6B is known to propagate preferentially in T lymphocytes or glial cells [49] and does not have any known oncogenes. The presence of HHV-6B in the colon and rectum may possibly originate from tissue infiltration by infected lymphocytes, although this hypothesis needs to be verified. One of the limitations of our study is the lack of availability of corresponding samples for further validation.

**Table 4:** Counts of colorectal samples, positive (+) or negative (−) for identified viruses, within tumor’s DNA/RNA pairs

**Sample type**	**Virus name**	**Tumor DNA (*N*** **= 117)**		**CI for two one-sided hypotheses (CL = 0.975)**	
Tumor RNA	EBV	+	−	0; 3.09	0.40; infinity
	+	15	9		
	−	10	83		
	CMV	+	−	0; 0.51*	0.01; infinity
	+	13	13		
	−	1	90		
	HHV-6B	+	−	0; 18.84	0.85; infinity
	+	2	3		
	−	10	102		
	HPV-18	+	−	0; 0.14*	0; infinity
	+	0	28		
	−	0	89		

*N*, number of matched pairs. *proportions are not equivalent at *α* = 0.05 after Bonferroni correction.

**Table 5:** Counts of colorectal samples, positive (+) or negative (−) for identified viruses, in matched tumor/normal specimen pairs

**Sample type**	**Virus name**	**Matched control DNA *N*** **= 111 2 (blood:solid)**		**Adjusted *p *** **values for association**		
				**Tumors vs. all controls**	**Tumors vs. blood**	**Tumors vs. solid tissue**
Tumor DNA	EBV	+	−	0.02	0.06	1
	+	3 (3:0)	21 (19:2)			
	−	5 (5:0)	82 (64:18)			
	CMV	+	−	0.03	0.06	1
	+	0	12 (11:1)			
	−	1 (1:0)	98 (79:19)			
	HHV-6B	+	−	1	0.98	0.07
	+	4 (1:3)	8 (8:0)			
	−	10 (2:8)	89 (80:9)			
	HPV-18	+	−	-	-	-
	+	0	0			
	−	0	0			

*N*, number of matched pairs. blood: solid, counts for the non-malignant samples by tissue type: whole blood and solid adjacent tissue, respectively.

### Virus integration into the human genome

**Figure 4:**
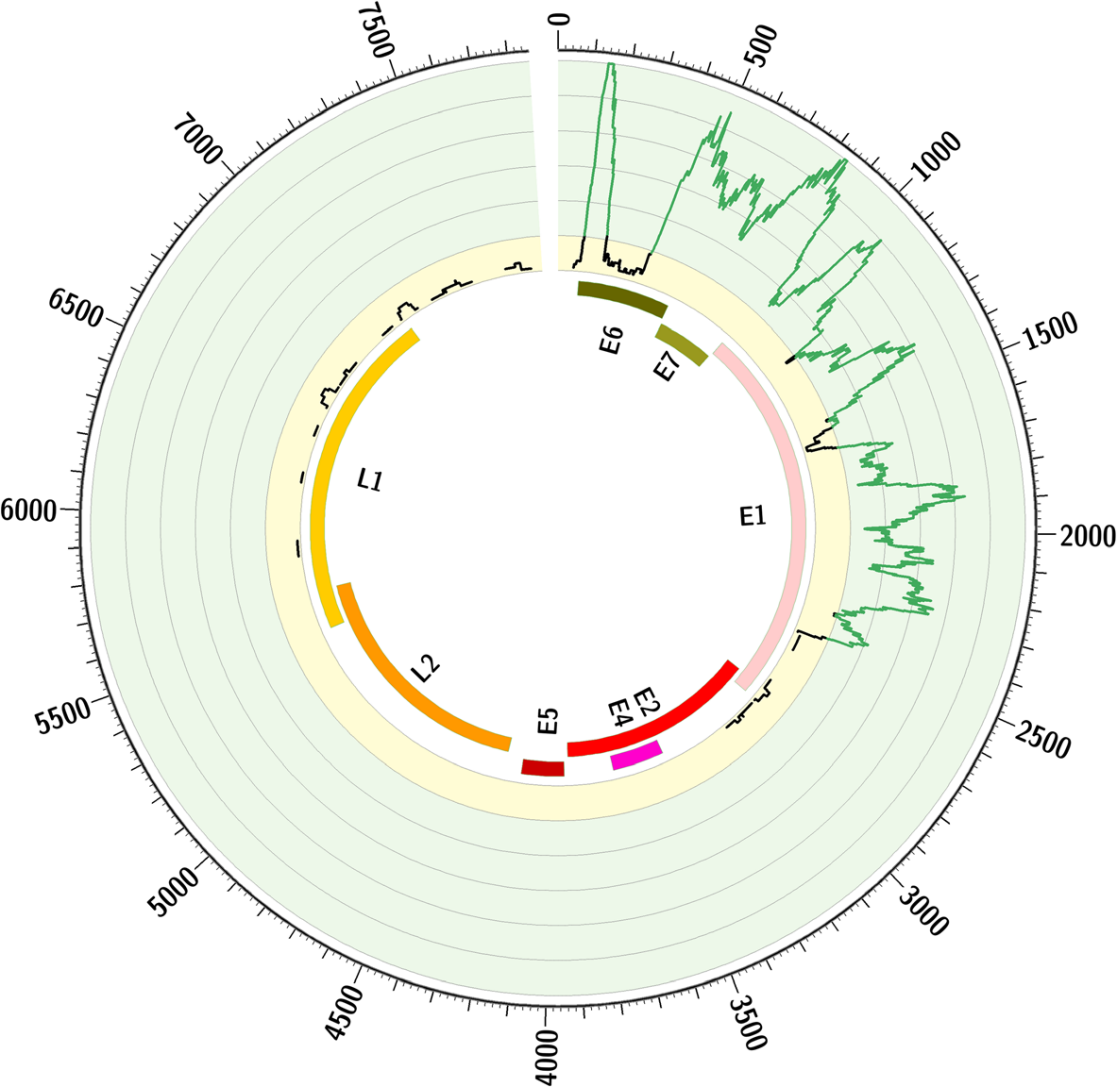
**Circos plot for HPV-18 RNA-seq.** From outside to the inside of the circle: (1) HPV-18 genomic positions; (2) number of sequencing reads mapped to the viral genome in 64 colon and rectum adenocarcinomas combined. Range from 1 to 10 shown on light yellow background as a black line, and from 11 to 61 on light green background as a green line; (3) viral genes mapped to genomic positions. While expression of genes *E6*, *E7*, and *E1* is obvious, genes *L2*, *E5*, *E4*, and part of *E2* were not detected. This pattern is expected when HPV-18 integrates in the host genome and part of the viral genome becomes deleted.

Since no viral DNA for HPV-18 was identifiable, detection of integration sites of HPV-18 into the human genome was not possible. Nevertheless, the cumulative expression pattern of HPV-18 in CRAD supports evidence for genomic integration. The HPV episome usually becomes integrated into the host cell DNA during oncogenesis by opening the ring molecule and disrupting the *E2* gene, which normally suppresses oncogenes *E6* and *E7.* As a result, part of *E2* and *L2* and whole *E4* and *E5* genes become deleted. Expression of *E6* and *E7* downregulates p53 and pRb and promotes malignancy [50]. While three early viral genes, *E1*, *E6*, and *E7*, showed high ‘cumulative expression levels’ in CRAD, partial expression of *E2* and whole *L2*, *E4* and *E5* was not detected (Figure 4). Lack of expression in presumably deleted regions suggests the potentially oncogenic nature of HPV-18. Despite collected evidence, the temporal relationship between infection and tumorigenesis cannot be disseminated from this data. To conclusively prove a causal role of viruses in cancer, a complete chain of evidence from epidemiology, histopathology, and molecular biology is required.

For the remaining viruses detected in available genomic data, the number of identified reads was not sufficient for integration site detection (see Figure 3 and Additional file 2). Neither viral nor human genomes were covered without substantial gaps. As shown in Table 2, the median coverage of the human genome in colorectal samples was below 2x, and a great majority of the viruses with available whole genome seq data had a small fraction of the genomes covered.

## Conclusions

Our results clearly demonstrate the presence of viral sequences in GIA. EBV and CMV were statistically significantly associated with CRAD. In addition, the expression pattern of HPV-18 was consistent with genomic integration typical during oncogenesis [50]. This supports the hypothesis that EBV, CMV, and HPV-18 are potentially oncogenic in GIA, although we realize that further studies are needed before a conclusion can be made about the pathophysiological role of the identified viruses in GIA. No viruses were identified in the remaining six cancer types.

Our results demonstrate the feasibility of NGS for the identification of viruses at very low levels in the human tissue. Unlike PCR-based approach, NGS data offer a unique opportunity to capture any viral nucleic acids present in the sample above the detection limit. Identification of viral infection is a first step in determining the role of viruses in cancer. Availability of comprehensive viral databases makes it possible to scan for a large number of candidates without the need for *de novo* assembly with the restriction that novel viruses will not be detected.

Finally, in this study, we established an empirical detection limit in our computational pipeline. This information can be used to calculate the required sequencing depth, as well as the amount of material needed by the given size of viral genome, and the expected viral load in similar studies.

## Methods

### Subjects

Whole transcriptome sequencing data for nine cancer types, comprising 1,007 patients, were obtained through TCGA (accessed on October 17, 2011). Table 1 summarizes specimen counts and abbreviations for the nine included cancer types. Additional transcriptome and whole genome sequencing data for three GIA were downloaded on November 1, 2012. Sequencing has been done using Illumina (Solexa, GAII, Illumina, Inc., San Diego, CA, USA) or SOLiD™ technology (Life Technologies, Carlsbad, CA, USA). A detailed description of the TCGA data can be found on the following TCGA websites: http://cancergenome.nih.gov/, https://tcga-data.nci.nih.gov/tcga/, as well as in two recently published studies on genomic characterization of three out of nine cancer types discussed here [10,11]. Patient enrollment and utilization of data were conducted in accordance with TCGA human subjects protection and data access policies (http://cancergenome.nih.gov/PublishedContent/Files/pdfs/6.3.1_TCGA_Human_Subjects_and_Data_Access_policies_FINAL_011211.pdf).

Cancers selected for the TCGA study were chosen based on specific criteria that included (1) poor prognosis and high public health impact, and (2) availability of human tumor and matched normal tissue that meet TCGA standards for patient consent, quality, and quantity. The proportion of 60% tumor nuclei in the specimens was found to be sufficient by TCGA project organizers to generate high-quality data, in which the tumor’s signal can be distinguished from other cells’ signals when using NGS. Only primary, untreated tumors were collected. Samples were frozen quickly after surgery in order to prevent degradation of the RNA and DNA.

Whole genome sequencing data was available for 37.5% of the GIA (Table 1). Blood or germ line specimens derived from the same individual as the tumor specimens were used in the TCGA study to serve as paired normal controls when available (Table 1). DNA sequencing data from the blood or adjacent healthy tissue was available for 35% of the GIA specimens.

### Bioinformatics analysis

Transcriptome data (BAM files) generated by TCGA for a total of 1,007 cancer specimens were analyzed in an automated fashion on a computational cluster hosted by the High-Performance Computing core at the Center for Computational Science, University of Miami (http://ccs.miami.edu/). An IBM BladeCenter cluster was available for compute-intensive data analysis. The cluster, named *Pegasus* running under Linux operation system, was used consisting of 280 computing nodes each with 8 Xeon 2.6 GHz cores and 16 GB of memory, and 700 computing nodes each with 4 Opteron 2.2 GHz core and 4 GB of memory. These nodes are interconnected by Gigabit Ethernet and feature a 21 TB NFS file system providing an aggregate of 5,040 cores and 7.3 TB of memory. All computational tasks were submitted in parallel to the LSF job scheduler and resource management system. The computational pipeline is outlined in Figure 1. In total, 1,156 jobs were submitted to the cluster for steps I–V for DNA-seq and RNA-seq data; 25,245 CPU hours were used for data analysis. Bamtools-1.0.2 [51] and samtools-0.1.18 [52] software were employed for converting data format. Sequencing reads with phred-like quality scores *q* > 30 were utilized. TopHat (v.2.0.0) [53] was consistently used for all transcriptome mapping steps. Multiple threads were used during alignment with option –p 8. When subtracting bacterial and viral sequences, we allowed TopHat to tolerate up to four mismatches per read, instead of the default of two in the alignment step, to allow for potentially higher mismatch rates due to mutations [54] or imperfect match to the reference sequence. In addition, TopHat was instructed to use a .gtf file and not to look for novel transcript junctions by utilizing the ‘—no-novel-junc’ flag. We combined reference fasta files into ‘supergenomes’ for vector sequences, bacterial genomes, and viral genomes for steps II, III, and IV, respectively (Figure 1). Each individual reference sequence in the ‘supergenome’ was treated as a chromosome. Reference files were indexed before alignment steps. The bacterial reference file had to be split into two parts to reduce the memory use needed for indexing and mapping steps. Even the single short read aligning to the viral reference was considered as successful detection, if following BLAST analysis [55] versus the NCBI nucleotide (nt) collection confirmed sequence similarity with the target over 98% and at least one transcriptome out of a cohort had more than 10 reads mapped to a viral genome reference. Sequences aligning to multiple organisms, known artificial (vector) sequences, or low-complexity sequences were considered false positive and removed.

Only three gastrointestinal cancer types, stomach (STAD), rectum (READ), and colon adenocarcinomas (COAD), which tested virus positive on the transcriptome level, proceeded to the whole genome analysis step (Table 1). Burrows-Wheeler Aligner (BWA, v.0.5.9) [56] with default options was consistently used for genomic data alignment. Multiple threads were used when running BWA with option –t 4. Genomic read subtraction was performed in exactly the same fashion as described above for transcriptomes (Figure 1). In order to determine computational pipeline sensitivity to single nucleotide mismatches, sequence reads on EBV and HPV-18 with different mutation/mismatch rates and lengths were simulated and run through the computational subtraction and alignment steps (see Additional files 1 and 4).

### Consensus sequence for EBV

EBV reference genomes include two very similar strains HHV-4.1 (NC_007605) and HHV-4.2 (NC_009334). In order to capture as many reads as possible, we created a consensus sequence using both NC_007605 and NC_009334 genome references. We replaced the two original references for two strains with the consensus reference for computational pipeline.

### Statistical analysis

Since available data comprised cases-only DNA/RNA and malignant/normal tissue pairs, a simple McNemar test implemented in R library ‘exact2x2’ (v1.1-1.0) was used for both equivalence and association testing [57]. Equivalence testing for virus identification in DNA and RNA was performed using two one-sided exact McNemar tests with a confidence level of 0.975 at *α* = 0.05 (Bonferroni-corrected for four viruses). The null hypothesis of equivalence was rejected, when at least in one of the one-sided tests, the confidence interval (CI) did not include ‘1’. Association of virus with tumor vs. normal tissue was done with two-sided exact McNemar test. Since COAD and READ are genetically identical [11], we combined two cohorts for association analysis to achieve a higher sample size. Association testing of virus presence in tumor/normal tissue was done using blood and solid tissue controls in separate tests, as well as blood and solid combined. Bonferroni correction was done for nine tests (three types of control groups multiplied by three viruses identified in the whole genome data). Fisher exact test was used for association testing with clinicopathological and demographics variables. Age at initial diagnosis in virus-positive and virus-negative groups was compared using ANOVA. Bonferroni correction was applied.

### Estimation of viral load

We calculated *V*, the number of viral copies per cell (vc/c), i.e., viral load, for each DNA sample as

$$V=C_{V}/C_{H},$$

where *C*_V_ is the average sequencing coverage for the virus, and *C*_H_ is the average human genome coverage. Average coverage *C* was calculated as

$$C_{i}=R_{i}\times L/G_{i},$$

where *i* is the species, *R* is the number of reads, *L* = 51 and is the average read length in nucleotides, and *G* is the corresponding genome length in nucleotides. For the diploid human genome, *C* = *C*_H_*/*2*.*

## Abbreviations

AML: Acute myeloid leukemia; CI: Confidence interval; CMV: Cytomegalovirus; COAD: Colon adenocarcinoma; CRAD: Colorectal adenocarcinomas; EBV: Epstein-Barr virus; GIA: Gastrointestinal adenocarcinomas; HHV: Human herpes virus; HHV-6B: Roseola virus; HPV-18: Human papilloma virus type 18; KIRK: Kidney renal clear carcinoma; KIRP: Kidney renal papillary cell carcinoma; KSHV: Kaposi’s sarcoma-associated human virus; LUAD: Lung adenocarcinoma; LUSK: Lung squamous cell carcinoma; NGS: Next-generation sequencing; nt: Nucleotide; READ: Rectum adenocarcinoma; SD: Standard deviation; STAD: Stomach adenocarcinoma; TCGA: The Cancer Genome Atlas; UCEC: Uterine corpus endometrioid carcinoma; vc/c: Viral copies per cell.

## Competing interests

The authors declare that they have no competing interests.

## Authors’ contributions

DS has designed and tested the analytical pipeline, downloaded and analyzed the data, and performed results interpretation and manuscript writing. NT has been involved in drafting and revising the manuscript and has contributed to the analytical pipeline design. Both authors have given final approval of the version to be published.

## Supplementary Material

Additional file 1 **Simulations and viral rate estimations.** A document containing simulation methods and results, and supplementary Tables 1–12, showing summary on viral reads detected, as well as clinical and demographic data in gastrointestinal adenocarcinomas. Click here for file

Additional file 2 **Dot matrix.** This dot matrix view shows regions of similarity based upon the BLASTN 2.2.27+ results. The viral genome positions are on the *X*-axis. The lines represent mapped reads. The *Y*-axis shows cumulative bases of the aligned reads over all GIA sorted by the percentage of the genome covered. Higher coverage yields longer lines on the plot. Viral RNA transcriptome reads, when mapped to their reference genome sequences, showed uneven distribution clustering most likely corresponding to actively transcribed genes. Genomic reads, as expected, mapped along the viral reference genome randomly and more uniformly than transcriptomic reads. Click here for file

Additional file 3 **Correlation of EBV and CMV load in tumor’s transcriptomes and genomes.** Each data point represents one tumor. The *X*-axis shows log10-transformed percentage of viral reads in tumor’s transcriptome; the *Y*-axis shows log10-transformed viral load (nc/c, see methods) in tumor’s whole genome. COAD are depicted as read circles, READ as blue triangles. STAD is not shown because there was not a sufficient number of tumor genomes sequenced. Click here for file

Additional file 4 **Simulation results.** At the low mutation rate up to 2%, derived reads were not lost to any significant extent, and our pipeline still captured over 94% simulated reads by BWA (whole genome pipeline) and over 80% by Tophat v.2.0.0 (transcriptome pipeline). Our approach had highest sensitivity with the shortest reads (50 nt), being at least 80% for BWA at a mismatch rate of 0.04, and 0.05 for Tophat. Higher mutation rates greatly impacted sensitivity, especially for the longer sequence reads, consistent with the BWA and TopHat algorithms. TopHat used the bowtie2 aligner, which seems to be affected to a greater extent by the length of the reads, probably due to using a fixed number of mismatches (*N* = 4), while BWA allows a floating error rate *k* depending on the read length. Simulated errors were randomly distributed. The longer the read, the more likely was the inclusion of mismatches. Subtraction of non-viral reads did not affect HPV-18 alignment, and less than 1% of CMV reads were lost through this process at zero mutation rate. As expected, computational subtraction eliminates individual viral sequences to various extents, depending on the degree of homology with non-viral reference sequences included in the filters. Click here for file
