# Supplementary material for: Viral expression associated with gastrointestinal adenocarcinomas in TCGA high-throughput sequencing data
Source: Hum Genomics. 2013 Nov 27;7(1):23. doi: 10.1186/1479-7364-7-23 (PMC3906926; doi:10.1186/1479-7364-7-23)
Supplement: Additional file 1 — Simulations and viral rate estimations. A document containing simulation methods and results, and supplementary Tables 1–12, showing summary on viral reads detected, as well as clinical and demographic data in gastrointestinal adenocarcinomas. [file 1479-7364-7-23-S1.docx]

**Simulations**

We simulated DNA sequencing fragments from the HPV-18 (NC_001357) and CMV (NC_006273) genomic reference sequences. These two viruses differ in genome length and number of genes. Single base mutations were introduced at different rates. Target mutation/mismatch rate (P) was introduced in the range from zero to ten percent, with a step of one percent. The genomic reference sequence in FASTA format was modified by simulation via random swapping of nucleotides with any of three alternatives, so that the target P can be achieved. The location of each nucleotide swap was drawn randomly from a uniform distribution. 100,000 single end sequencing reads of length 50, 100 and 150 nucleotides were simulated from the modified reference sequences that were created in the previous step and saved in FASTQ-format [[1](#ENREF_1)]. Finally, simulated fragments underwent computational subtraction and mapping to the viral reference sequences in the same manner as TCGA data.

**Viral mutation rate estimation**

Unfortunately, exact mismatch rate between the reference sequence and the sample sequence is not known for a majority of viruses. The mutation rates in double-stranded DNA (sdDNA) viruses, to which HHVs and HPVs belong, tend to be lower than in RNA viruses and are estimated to be around 10^-8^-10^-7^ substitutions per nucleotide per cell (s/n/c) [[2](#ENREF_2)] and negatively correlate with the genome size. Intestinal epithelial cells proliferate and turn over very quickly [[3](#ENREF_3)], and it may take years from viral infection to onset of malignancy. If we assume that the CMV virus has a similar mutation rate as that of Herpes Simplex (5.9x10^-8^ s/n/c) and gut cells proliferate every 24 hours, we can calculate its mutation rate MR over 10,000 cell cycles (over 27.4 years) as:

MR=(5.9 x 10^-8^ x N x 10,000)/N = 0.0059;

where the genome length N is 235,646 nt. Note that these calculations do not take into account selective pressure.

Our approach was sensitive to the read length and mismatch rate (additional figure 3). Natural mutation rates for dsDNA viruses are low [[2](#ENREF_2)] and do not seem to impose an issue for detection in our pipeline, however, it may be more critical for viruses with higher mutation rates or with incomplete/mismatching reference sequence.

**Literature**

1. Cock PJ, Fields CJ, Goto N, Heuer ML, Rice PM: The Sanger FASTQ file format for sequences with quality scores, and the Solexa/Illumina FASTQ variants. Nucleic Acids Res 2010, 38(6):1767-1771.

2. Sanjuan R, Nebot MR, Chirico N, Mansky LM, Belshaw R: Viral mutation rates. Journal of virology 2010, 84(19):9733-9748.

3. Creamer B, Shorter RG, Bamforth J: The turnover and shedding of epithelial cells. I. The turnover in the gastro-intestinal tract. Gut 1961, 2:110-118.

**Figure legends**

**Additional Figure 1: Dot matrix (Additional file 2)**

This dot matrix view shows regions of similarity based upon the BLASTN 2.2.27+ results. The viral genome positions are on the X-axis. The lines represent mapped reads. Y-axis shows cumulative bases of the aligned reads over all GIA sorted by the percentage of the genome covered. Higher coverage yields longer lines on the plot. Viral RNA transcriptome reads, when mapped to their reference genome sequences, showed uneven distribution clustering most likely corresponding to actively transcribed genes. Genomic reads, as expected, mapped along the viral reference genome randomly and more uniformly than transcriptomic reads.

**Additional Figure 2: Correlation of EBV and CMV load in tumor’s transcriptomes and genomes (Additional file 3)**

Each data point represents one tumor. X-axis shows log10-transformed percentage of viral reads in tumor’s transcriptome, Y-axis shows log10-transformed viral load (nc/c, see methods) in tumor’s whole genome. COAD are depicted as read circles, READ as blue triangles. STAD is not shown, because there was not a sufficient number of tumor genomes sequenced.

**Additional Figure 3**: **Simulation results (Additional file 4)**

At the low mutation rate up to 2%, derived reads were not lost to any significant extent and our pipeline still captured over 94% simulated reads by BWA (whole genome pipeline) and over 80% by Tophat v.2.0.0 (transcriptome pipeline). Our approach had highest sensitivity with the shortest reads (50 nt), being at least 80% for BWA at a mismatch rate of 0.04, and 0.05 for Tophat. Higher mutation rates greatly impacted sensitivity, especially for the longer sequence reads, consistent with the BWA and Tophat algorithms. Tophat used bowtie2 aligner, which seems to be affected to a greater extent by the length of the reads, probably due to using a fixed number of mismatches (N=4), while BWA allows a floating error rate “k” depending on the read length. Simulated errors were randomly distributed. The longer the read, the more likely was the inclusion of mismatches. Subtraction of non-viral reads did not affect HPV-18 alignment, and less than 1% of CMV reads were lost through this process at zero mutation rate. As expected, computational subtraction eliminates individual viral sequences to various extent, depending on the degree of homology with non-viral reference sequences included in the filters.

**Additional Table 1: Summary on numbers and proportions of viral reads, median / average, detected in gastrointestinal cancers**

| **Data source** | **Statistics** | **EBV** | **CMV** | **HHV-6B** | | **KSHV '** | | **HPV-18** |
| --- | --- | --- | --- | --- | --- | --- | --- | --- |
| Whole transcriptome (RNA) | Number of mapped reads | 3 / 526.5 | 9 / 45.1 | | 7 / 58.8 | | 115 | 6 / 14.8 |
|  | % of viral reads | 8.56×10^-6^ / 3.88×10^-4^ | 2.58×10^-5^ / 9.52×10^-5^ | | 1.33×10^-5^ / 2.04×10^-4^ | | 7.27×10^-5^ | 2.54×10^-5^/ 7.24×10^-5^ |
| Whole genome (DNA) | Number of mapped reads | 2 / 10.6 | 3 / 14.8 | | 8 / 357.6 | | NA | NA |
|  | Viral load (vc/c)* | 4.90×10^-4^ / 2.17×10^-3^ | 2.20×10^-3^ / 5.03×10^-4^ | | 1.57×10^-3^ / 5.96×10^-2^ | | NA | NA |

" * " We calculated the number of viral copies per cell (vc/c) (viral load) as the ratio of average sequencing coverage for the virus to the average human genome coverage for each sample. Average coverage was calculated as number of reads multiplied by the average read length (51 nt), divided by the corresponding genome length and divided by two for diploid human genome

"NA" - no data available

" ' " - Only one tumor sample was positive for KSHV

**Additional Tables 2-12: Contingency tables and summary statistics for virus association with clinical and demographics data in patients with colorectal cancer.**

“**Total N**” – number of patients with available clinical or demographic data

“**p-values**” – nominal p-values for association tests with virus positivity.

**Additional table 2: Anatomic subdivisions**

| **Group** | **Cecum** | **Ascending Colon** | **Hepatic Flexure** | **Transverse Colon** | **Splenic Flexure** | **Descending Colon** | **Sigmoid Colon** | **Rectosigmoid Junction** | **Rectum** | **p-value** |
| --- | --- | --- | --- | --- | --- | --- | --- | --- | --- | --- |
| Total N | 40 | 36 | 12 | 17 | 2 | 7 | 75 | 2 | 68 | - |
| HPV-18 | 18 | 11 | 3 | 4 | 1 | 1 | 21 | 0 | 3 | 5.00E-05 |
| EBV | 13 | 7 | 2 | 1 | 0 | 0 | 19 | 0 | 22 | 0.22 |
| CMV | 9 | 4 | 7 | 1 | 0 | 2 | 15 | 0 | 16 | 0.06 |
| HHV-6B | 2 | 5 | 3 | 0 | 0 | 0 | 8 | 2 | 5 | 0.02 |

**Additional Table 3: Histological type**

| **Group** | **Adenocarcinoma** | **Mucinous Adenocarcinoma** | **p-value** |
| --- | --- | --- | --- |
| Total N | 226 | 33 | - |
| HPV-18 | 58 | 6 | 0.39 |
| EBV | 57 | 6 | 0.51 |
| CMV | 45 | 7 | 0.81 |
| HHV-6B | 24 | 2 | 0.54 |

**Additional table 4: History of colon polyps**

| **Group** | **Yes** | **No** | **p-value** |
| --- | --- | --- | --- |
| Total N | 115 | 139 | - |
| HPV-18 | 38 | 23 | 0.003 |
| EBV | 30 | 34 | 0.77 |
| CMV | 28 | 25 | 0.22 |
| HHV-6B | 10 | 14 | 0.83 |

**Additional Table 5: Pathologic M**

| **Group** | **M0** | **M1** | **p-value** |
| --- | --- | --- | --- |
| Total N | 221 | 39 | - |
| HPV-18 | 54 | 8 | 0.84 |
| EBV | 57 | 7 | 0.32 |
| CMV | 49 | 4 | 0.20 |
| HHV-6B | 22 | 4 | 1 |

**Additional Table 6: Pathologic N**

| **Group** | **N0** | **N1** | **N2** | **p-value** |
| --- | --- | --- | --- | --- |
| Total N | 160 | 56 | 49 | - |
| HPV-18 | 41 | 14 | 9 | 0.59 |
| EBV | 47 | 9 | 10 | 0.10 |
| CMV | 33 | 14 | 8 | 0.58 |
| HHV-6B | 15 | 9 | 2 | 0.13 |

**Additional Table 7: Pathologic T**

| **Group** | **T1** | **T2** | **T3** | **T4** | **p-value** |
| --- | --- | --- | --- | --- | --- |
| Total N | 11 | 54 | 177 | 23 | - |
| HPV-18 | 2 | 14 | 39 | 9 | 0.32 |
| EBV | 4 | 20 | 40 | 2 | 0.03 |
| CMV | 1 | 18 | 32 | 4 | 0.09 |
| HHV-6B | 0 | 3 | 21 | 2 | 0.48 |

**Additional Table 8: Stage**

| **Group** | **I** | **II** | **III** | **IV** | **p-value** |
| --- | --- | --- | --- | --- | --- |
| Total N | 54 | 101 | 68 | 40 | - |
| HPV-18 | 12 | 27 | 15 | 9 | 0.89 |
| EBV | 23 | 23 | 13 | 7 | 0.01 |
| CMV | 16 | 17 | 17 | 5 | 0.12 |
| HHV-6B | 3 | 12 | 7 | 3 | 0.63 |

**Additional Table 9: Race**

| **Group** | **N** |
| --- | --- |
| ASIAN | 1 |
| BLACK OR AFRICAN AMERICAN | 6 |
| WHITE | 36 |
| No data available | 222 |

**Additional Table 10: Ethnicity**

| **Group** | **N** |
| --- | --- |
| Not Hispanic | 42 |
| No data available | 223 |

**Additional Table 11: Gender**

| **Group** | **Male** | **Female** | **p-value** |
| --- | --- | --- | --- |
| No data | 132 | 133 | - |
| HPV-18 | 32 | 32 | 1 |
| EBV | 32 | 34 | 1 |
| CMV | 28 | 27 | 1 |
| HHV-6B | 11 | 15 | 0.53 |

**Additional Table 12: Age at initial diagnosis.**

| Virus | Min. | 1^st^ Qu. | Median | Mean | 3^rd^ Qu. | Max. | p-value |
| --- | --- | --- | --- | --- | --- | --- | --- |
| All | 34 | 62 | 70 | 68.98 | 78 | 90 | - |
| HPV-18 | 34 | 63 | 72.5 | 70.58 | 80 | 90 | 0.22 |
| EBV | 48 | 64.25 | 69.5 | 69.82 | 77 | 89 | 0.51 |
| CMV | 36 | 65.5 | 72 | 71.6 | 80 | 89 | 0.07 |
| HHV-6B | 47 | 63.5 | 74.5 | 71.46 | 79.75 | 90 | 0.26 |
