# Supplementary material for: Viral expression associated with gastrointestinal adenocarcinomas in TCGA high-throughput sequencing data
Source: Hum Genomics. 2013 Nov 27;7(1):23. doi: 10.1186/1479-7364-7-23 (PMC3906926; doi:10.1186/1479-7364-7-23)

EBV, NC_007605

Transcriptome

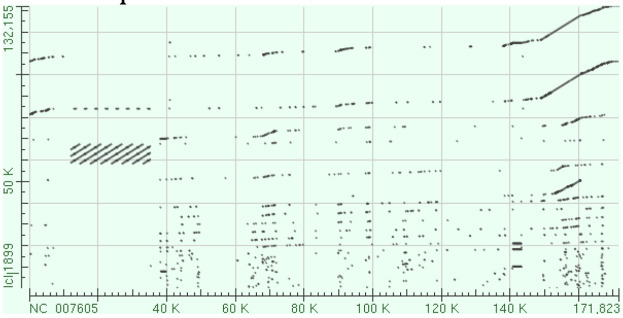

Genome

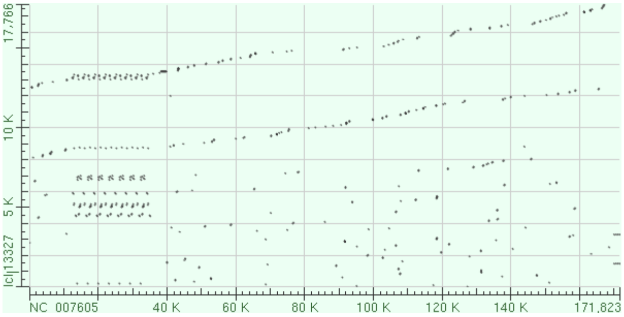

CMV, NC_006273

Transcriptome

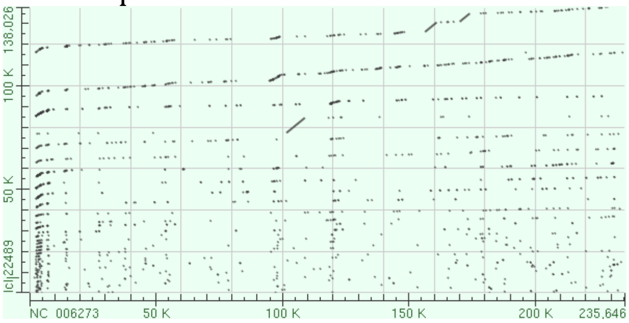

Genome

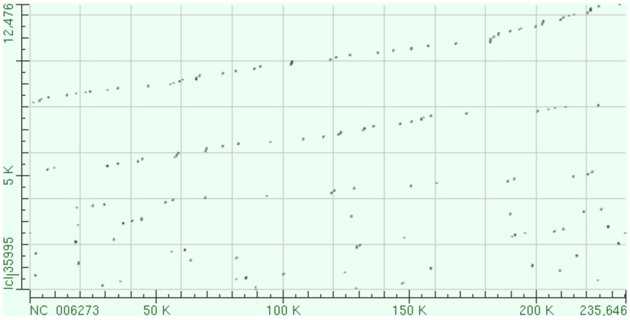

HHV-6B, NC_000898

Transcriptome

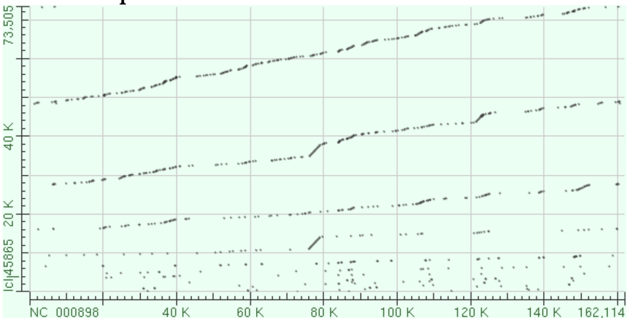

Genome

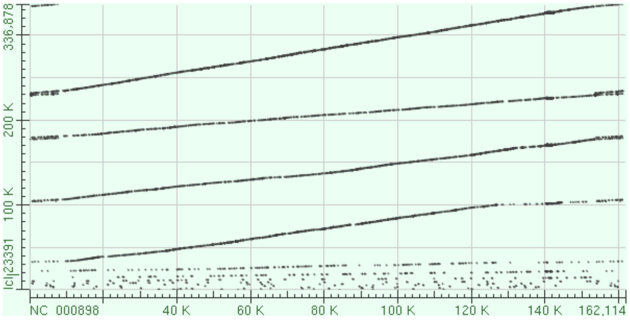

KSHV, NC_009333

Transcriptome

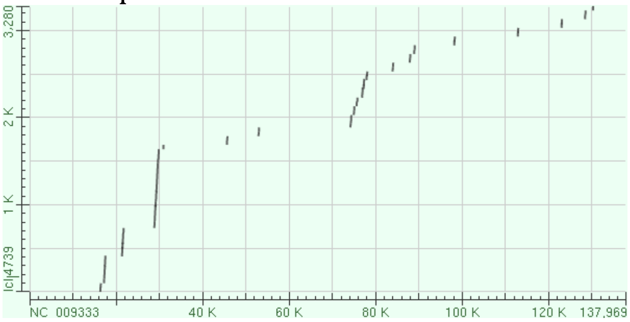

HPV-18, NC_001357

Transcriptome

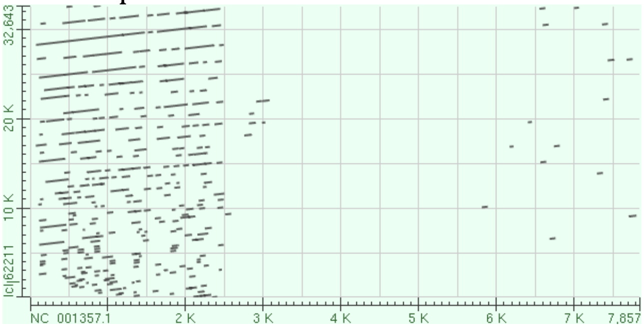

Supplement: Additional file 2 — Dot matrix. This dot matrix view shows regions of similarity based upon the BLASTN 2.2.27+ results. The viral genome positions are on the X-axis. The lines represent mapped reads. The Y-axis shows cumulative bases of the aligned reads over all GIA sorted by the percentage of the genome covered. Higher coverage yields longer lines on the plot. Viral RNA transcriptome reads, when mapped to their reference genome sequences, showed uneven distribution clustering most likely corresponding to actively transcribed genes. Genomic reads, as expected, mapped along the viral reference genome randomly and more uniformly than transcriptomic reads. [file 1479-7364-7-23-S2.pdf]
