# Supplementary figures and images for: Viral expression associated with gastrointestinal adenocarcinomas in TCGA high-throughput sequencing data
Source: Hum Genomics. 2013 Nov 27;7(1):23. doi: 10.1186/1479-7364-7-23 (PMC3906926; doi:10.1186/1479-7364-7-23)

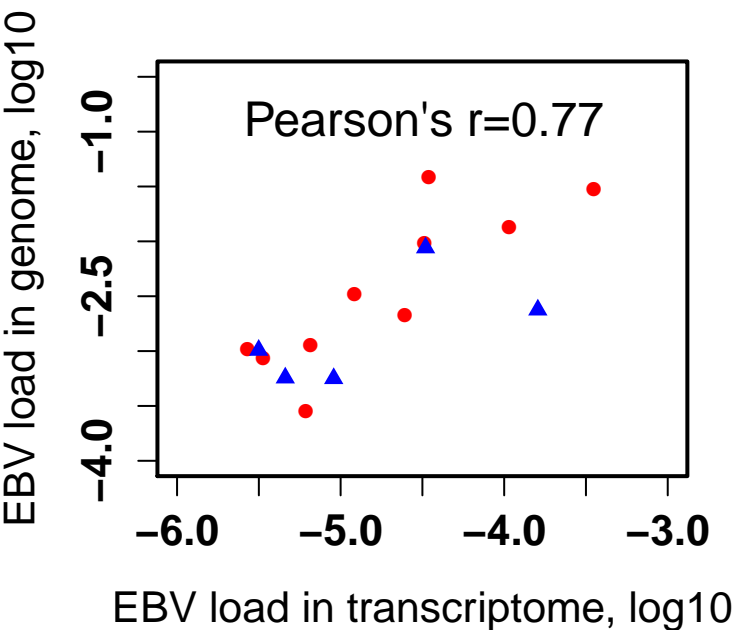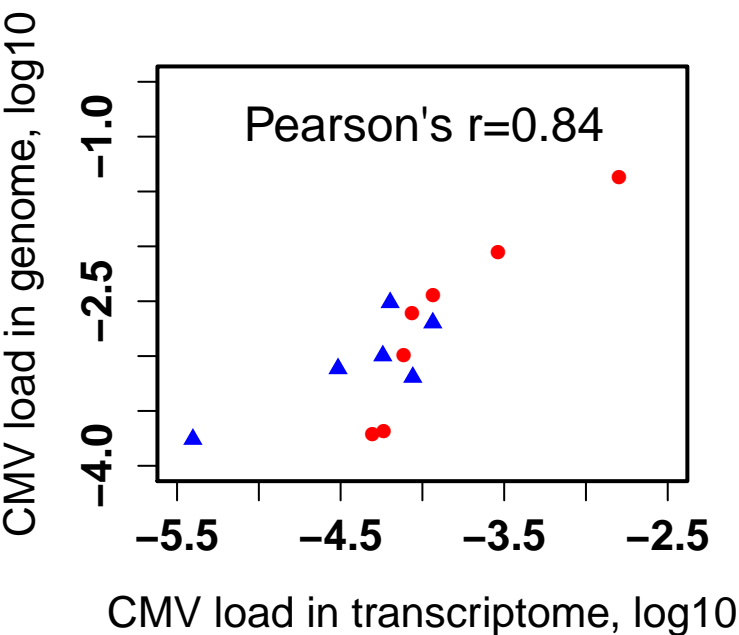

Supplement: Additional file 3 — Correlation of EBV and CMV load in tumor’s transcriptomes and genomes. Each data point represents one tumor. The X-axis shows log10-transformed percentage of viral reads in tumor’s transcriptome; the Y-axis shows log10-transformed viral load (nc/c, see methods) in tumor’s whole genome. COAD are depicted as read circles, READ as blue triangles. STAD is not shown because there was not a sufficient number of tumor genomes sequenced. [file 1479-7364-7-23-S3.pdf]

BWA

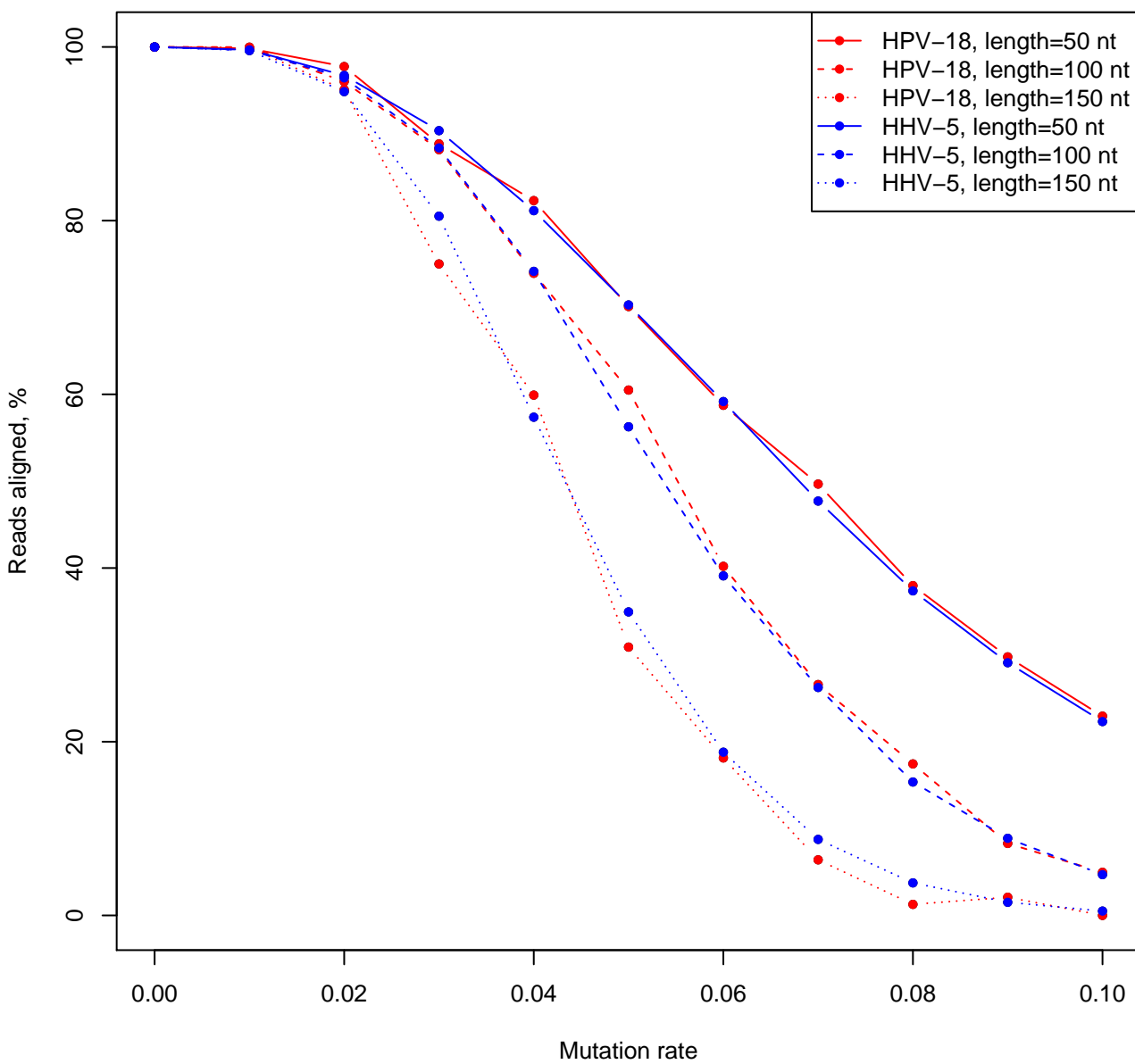

BWA

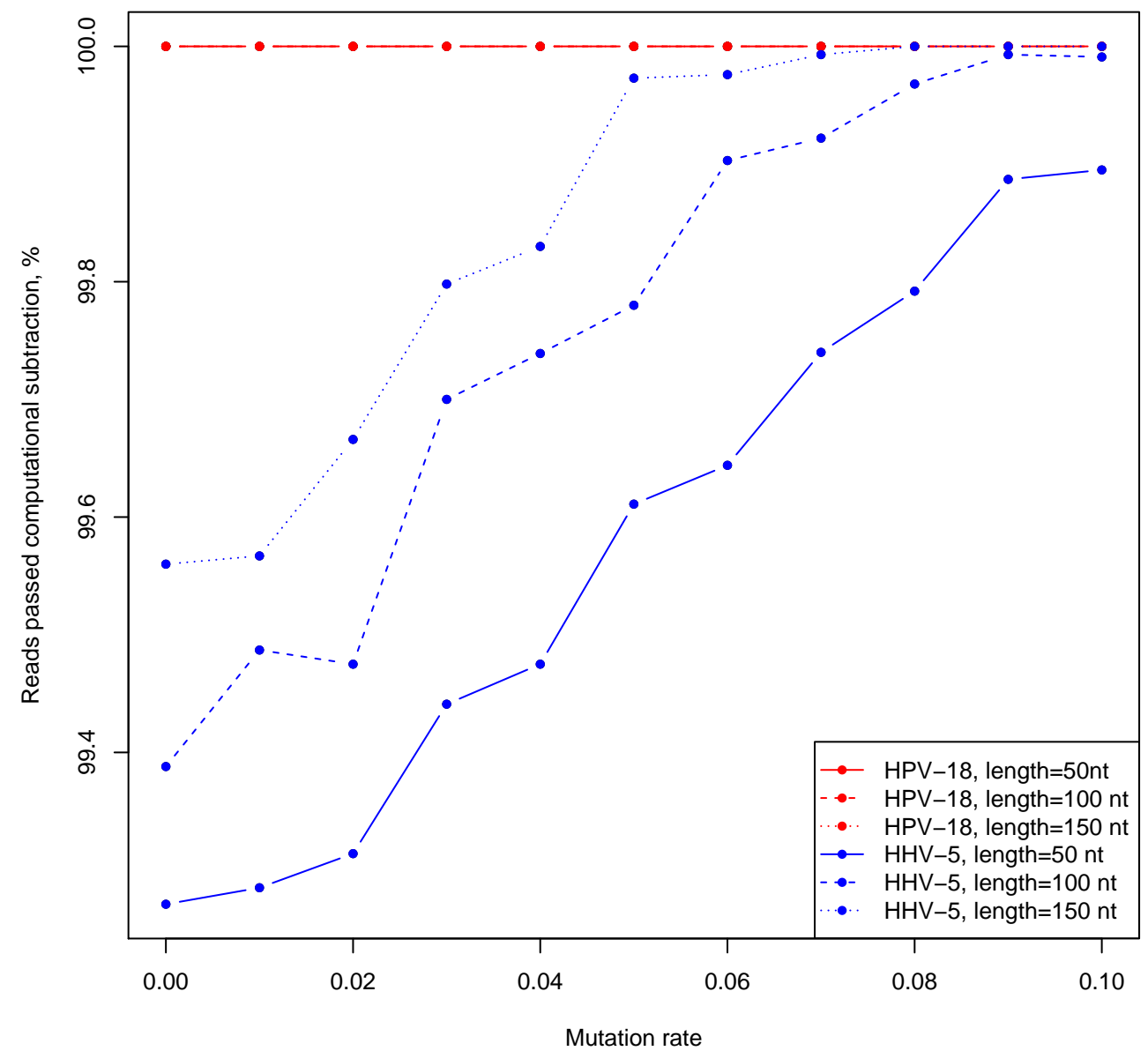

TopHat

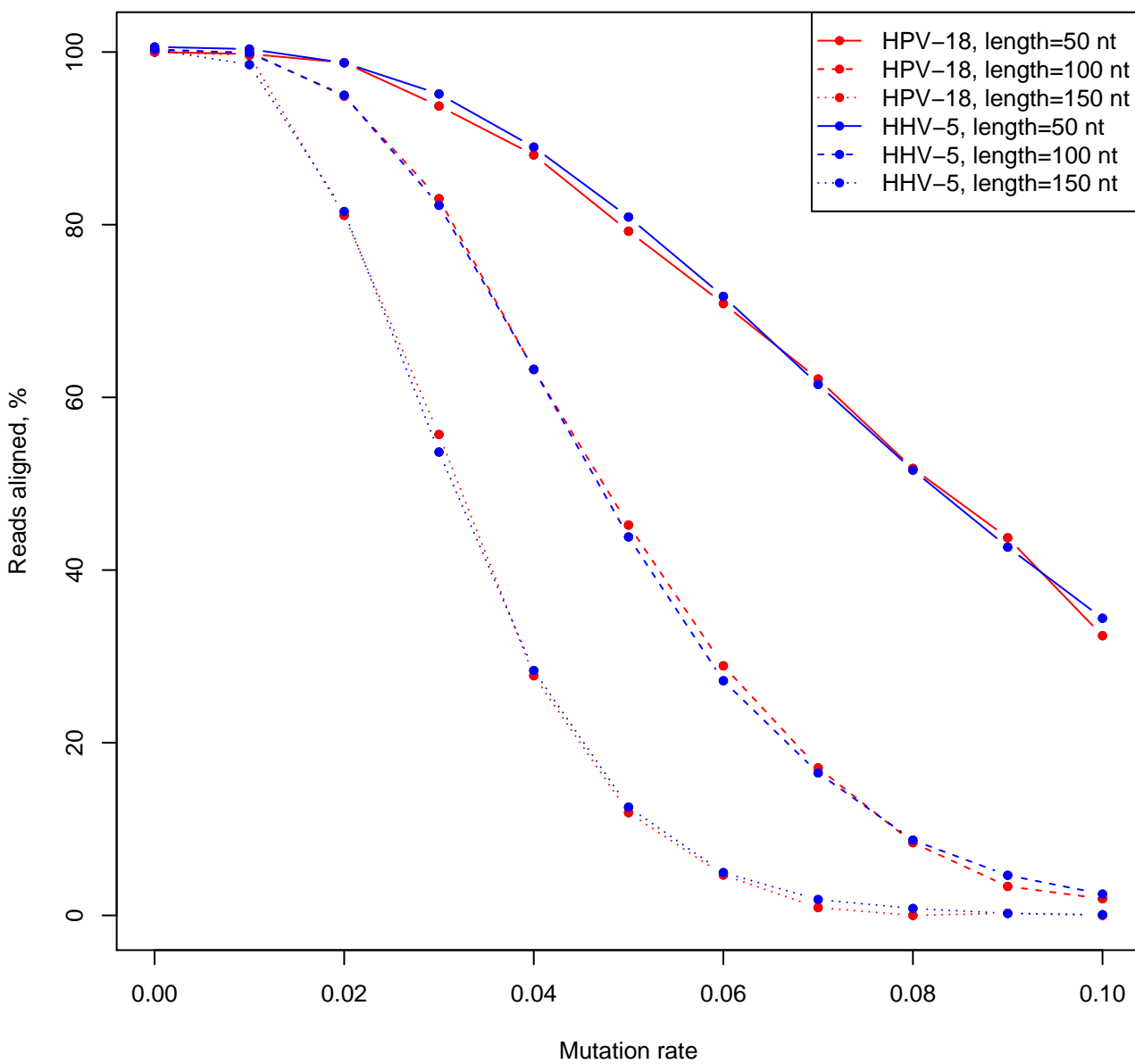

TopHat

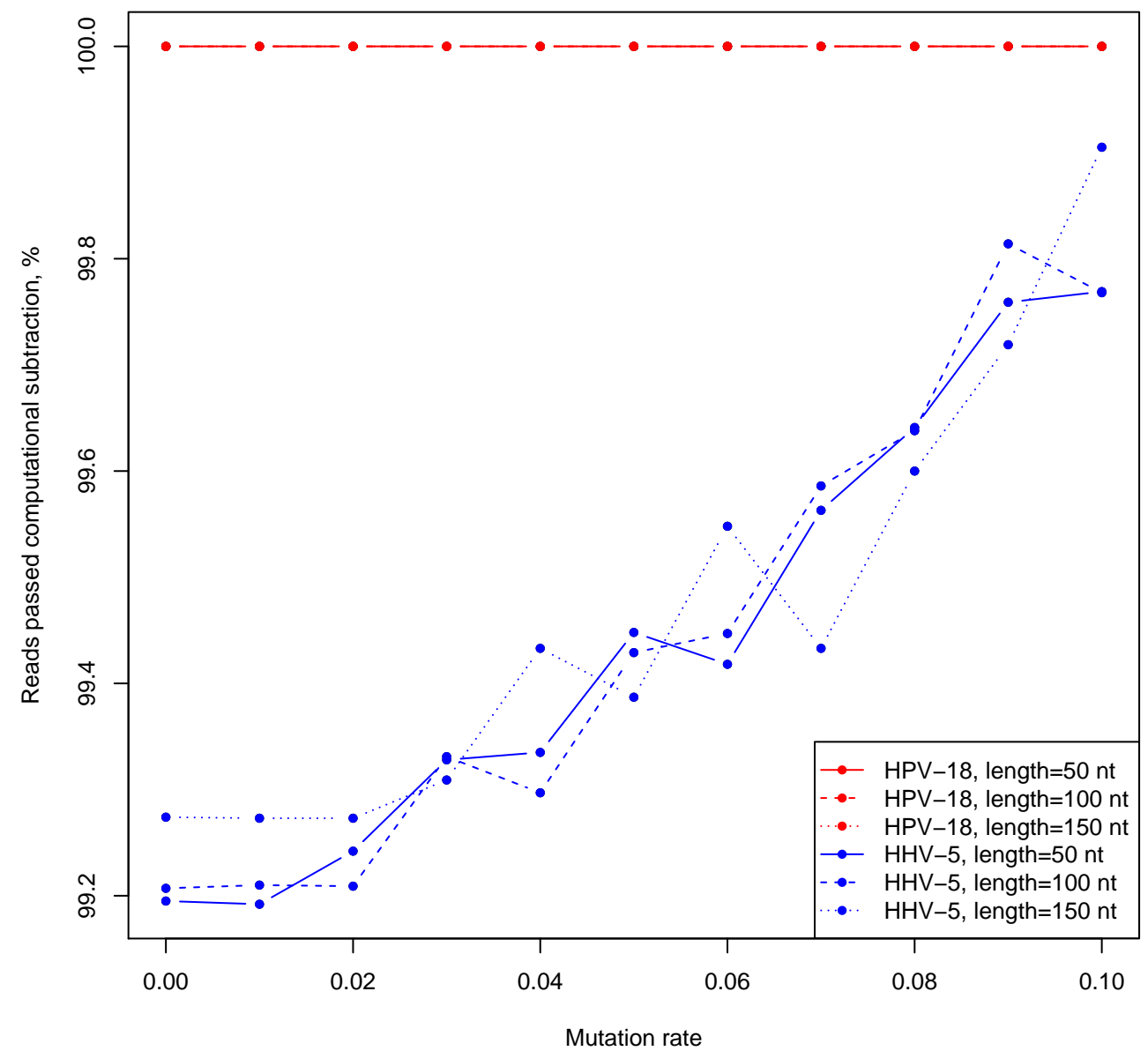

Supplement: Additional file 4 — Simulation results. At the low mutation rate up to 2%, derived reads were not lost to any significant extent, and our pipeline still captured over 94% simulated reads by BWA (whole genome pipeline) and over 80% by Tophat v.2.0.0 (transcriptome pipeline). Our approach had highest sensitivity with the shortest reads (50 nt), being at least 80% for BWA at a mismatch rate of 0.04, and 0.05 for Tophat. Higher mutation rates greatly impacted sensitivity, especially for the longer sequence reads, consistent with the BWA and TopHat algorithms. TopHat used the bowtie2 aligner, which seems to be affected to a greater extent by the length of the reads, probably due to using a fixed number of mismatches (N = 4), while BWA allows a floating error rate k depending on the read length. Simulated errors were randomly distributed. The longer the read, the more likely was the inclusion of mismatches. Subtraction of non-viral reads did not affect HPV-18 alignment, and less than 1% of CMV reads were lost through this process at zero mutation rate. As expected, computational subtraction eliminates individual viral sequences to various extents, depending on the degree of homology with non-viral reference sequences included in the filters. [file 1479-7364-7-23-S4.pdf]
